# Ultra-Wideband Microwave Imaging System for Root Phenotyping

**DOI:** 10.3390/s22052031

**Published:** 2022-03-05

**Authors:** Xiaodong Shi, Jiaoyang Li, Saptarshi Mukherjee, Srijan Datta, Vivek Rathod, Xinyu Wang, Wei Lu, Lalita Udpa, Yiming Deng

**Affiliations:** 1 Department of Electrical and Computer Engineering, Michigan State University, East Lansing, MI 48824, USA; shixiaod@msu.edu (X.S.); lijiao@msu.edu (J.L.); dattasri@egr.msu.edu (S.D.); udpal@egr.msu.edu (L.U.); 2 Lawrence Livermore National Laboratory, Livermore, CA 94550, USA; saptarshicity@gmail.com; 3 Oak Ridge National Laboratory, Oak Ridge, TN 37830, USA; tanuvivek@gmail.com; 4 College of Life Science, Nanjing Agricultural University, Nanjing 210095, China; xywang@njau.edu.cn (X.W.); njaurobot@njau.edu.cn (W.L.)

**Keywords:** in situ root measurement, ultra-wideband antenna, time reversal, microwave imaging, phenotyping, non-destructive inspection

## Abstract

The roots are a vital organ for plant growth and health. The opaque surrounding environment of the roots and the complicated growth process means that in situ and non-destructive root phenotyping face great challenges, which thus spur great research interests. The existing methods for root phenotyping are either unable to provide high-precision and high accuracy in situ detection, or they change the surrounding root environment and are destructive to root growth and health. Thus,we propose and develop an ultra-wideband microwave scanning method that uses time reversal to achieve in situ root phenotyping nondestructively. To verify the method’s feasibility, we studied an electromagnetic numerical model that simulates the transmission signal of two ultra-wideband microwave antennas. The simulated signal of roots with different shapes shows the proposed system’s capability to measure the root size in the soil. Experimental validations were conducted considering three sets of measurements with different sizes, numbers and locations, and the experimental results indicate that the developed imaging system was able to differentiate root sizes and numbers with high contrast. The reconstruction from both simulations and experimental measurements provided accurate size estimation of the carrots in the soil, which indicates the system’s potential for root imaging.

## 1. Introduction

As one of the plant’s three major organs, the root system provides functions that are central to plant fitness, such as nutrient absorption, fixation, water transmission, synthesis and storage [1]. The spatial distribution of roots in the soil directly affects the growth and health of plants. In addition, root systems transfer carbon, which is captured from the atmospheric CO_2_ by plants, to soil and aid long-term soil carbon storage [2]. A better understanding of root phenotype in situ and non-destructively is important for the research of soil and plant science, earth system science and others.

Recently, many state-of-the-art research works have been conducted for structural health monitoring and fault diagnosis in the field of nondestructive evaluation (NDE) and have made remarkable progress [3,4], particularly, on plant’s aerial part structures and functions [5,6,7]. However, because the root system generally grows in dense, opaque soil, it is difficult to observe visually or optically [8,9,10].

Since 1727, traditional root phenotyping methods have typically been destructive, time- consuming and low-resolution, such as excavation methods [11], the pinboard method [12] and the trench profile technique [9]. The root imaging method became the main root phenotype method since the introduction of the glass pane method in 1873 [13], which is achieved by a researcher manually observing and drawing the root color, size and shape and cannot phenotype the root growth accurately. Bates [14] updated the glass pane to glass tube method to better apply it to in situ root phenotyping.

Neutron radiography was proposed and applied to obtain root growth images [15] starting in 1985. However, the long-term radiation effects on the plant root growth and the complexity and inconvenience of the equipment makes the neutron radiography method inapplicable for in situ root phenotyping. Since 2000, high-precision optical instruments and digital imaging methods have greatly improved the accuracy of root imaging technology and made non-destructive root phenotyping possible. X-ray computed tomography uses high energy photons to scan the root and reconstructs the root image using the decayed X-ray signals detected by the detector [16]. The X-ray computed tomography method can achieve non-destructive, high resolution, high accuracy and fast 3D root phenotyping [17,18,19].

However, the expensive and non-portable nature of X-rays makes this method ineffective for in situ root phenotyping. In addition, X-ray computed tomography is limited in recording root growth [20]. The Magnetic Resonance (MR) method has similar advantages and disadvantages to the X-ray computed tomography method, except for the lower resolution and shorter imaging time [21]. MR imaging is mainly dependent on the water content of the root, and thus its accuracy may be influenced by the plant type and soil moisture [22] in root imaging.

Computed tomography (CT) and position emission tomography (PET) have ionization radiation and can alter root development [23]. Laser root scanner can provide precise 3D measurements non-destructively; however, it is time-consuming and expensive [24]. According to the characteristics of the laser, it can only be used when the root is growing in a transparent medium.

Confocal laser scanning microscopy [25], cameras [26,27], fluorescence techniques [28,29] and the hyperspectral imaging method [9] are all limited to real soil as the laser scanner is. The non-invasive and non-contact thermoacoustic sensing and characterization of plants is ongoing research, which is still limited by the use of agarose [30]. The advantages and limits of the pre-existing root phenotype methods are summarized in Table 1.

Terahertz (THz) imaging is a powerful technique for the subsurface imaging of objects and roots in soil [31]. The applicability in the field is limited as its wireless band exceeds the 802.11 b protocol. Scattering, absorption and lesser penetration depth are additional hurdles in the implementation. To date, the unavailability of sources, detectors and modulators at affordable prices means this technology is generally unavailable to the users commercially. The microwave imaging method is a promising nondestructive evaluation technique that can provide a quantitative measure of the lossless or low-loss dielectric materials profile.

It has shown great promise in a wide range of applications, including but not limited to the imaging of composite structures [32] and low-dielectric-contrast media [33]. The Microwave NDE method has been used for testing voids, delamination, porosity etc. in dielectric materials, including polymers, ceramics, plastics and their composites [34].

Currently, microwave sensors, such as split-ring resonators [35] and metamaterial-based sensors [36], have shown great sensitivity for measuring the dielectric constant. However, these methods are typically used for fixed position detection and lack imaging ability. Microwaves have been also used in determining soil moisture in rhizoboxes [37]. A microwave resonator system was verified to have the ability to determine the plant biomass non-invasively [38], while it lacks the ability of root phenotyping. Non-contact inspection and the ability to penetrate dielectric materials are two of the most important microwave NDE attributes, which make it suitable for the in situ real-time monitoring of plant roots. Therefore, a microwave scanning method that uses Time Reversal (TR) is investigated for its potential application as a real-time and in situ root reconstruction imaging method.

In this paper, an ultra-wideband microwave imaging (UBMI) system is proposed and developed to offer low cost, high-contrast and fast NDE for plant root imaging. The system is capable of creating a dielectric map of the scanning area by extracting the changes in both magnitude and phase in the transmitted and reflected signal. The time-reversal method was used to create a 2D reconstruction image of the root, which can provide the size and position information of the root. The paper is organized as follows. Section 2 describes the time-reversal microwave microscopic imaging method and its capabilities. Section 3 estimates the size and location of the carrot using the UBMI system. Finally, our conclusions and future work are presented in Section 4.

## 2. Imaging System

**Figure 1:**
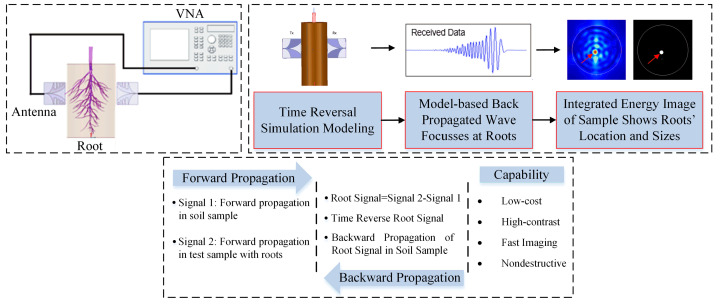
Flow chart of the root imaging technique using time reversal.

An ultra-wideband TR-based microwave imaging system was developed that offers low cost, high-contrast and fast NDE techniques for plants. The flow chart of the UBMI system is shown in Figure 1. A Vivaldi antenna array operating at ultra-wideband frequency from 3 to 10 GHz and providing high gain and symmetric beam-patterns was designed in accordance with [39] to detect roots buried in the soil in situ based on the properties of TR.

An arch range was constructed for fitting the antenna system with a fixed position transmitter antenna and a receiver antenna that is rotated around the scanned target with uniform angular steps to emulate an antenna array. A vector network analyzer (Keysight E8363B) was utilized to provide an excitation signal to the transmitting antenna that is radiated and transmitted through the root buried in the soil and recorded by the receiving antenna. Both the magnitude and phase measurements vary depending on the wave scattered by the soil and root sections. The perturbation due to the roots was computed and processed using a TR algorithm to image the root buried in the soil.

### 2.1. Time Reversal Imaging

The proposed TR algorithm offers a non-iterative processing framework for rapid root imaging. Unlike regularization based imaging techniques that rely on an iterative framework for inversion, TR employs a physics-based direct back-propagation technique to perform imaging. The method exploits the time symmetric nature of the scalar electromagnetic wave equation, expressed as

(1) $$\begin{array}{c} \begin{array}{c} \left(\nabla^{2}-\mu \varepsilon \frac{\partial^{2}}{\partial t^{2}}\right)\varphi \left(r,t\right)=0. \end{array} \end{array}$$

where *c* is the speed of light in free space and μ,ε are the material permeability and permittivity, respectively. The time-symmetric nature of the wave equation allows for fields diverging away to be reversed in time and converged back spatio-temporally to the scattering sources [40]. The detailed theory, numerical implementation and analysis of the TR algorithm was performed previously by the authors in [41]. As seen in [42], the TR algorithm can be implemented to detect targets and other scattering sources. The time-integrated energy (Θ) of the time reversed wave can be utilized to obtain a focused spatio-temporal image of the imaging domain as given by

(2) $$\Theta \left(x,y\right)=\int_{0}^{T}h_{n}^{2}\left(t\right)=\int_{0}^{T}{\left|E_{z}\left(x,y,t\right)\right|}^{2}dt,$$

where $h_{n}\left(t\right)$ and $h_{n}\left(-t\right)$ are the estimated forward and backward medium responses, and *T* is the total time. Here, $TM_{z}$ polarization is assumed, with $E_{x},E_{y}$ and $H_{z}=0$.

**Figure 2:**
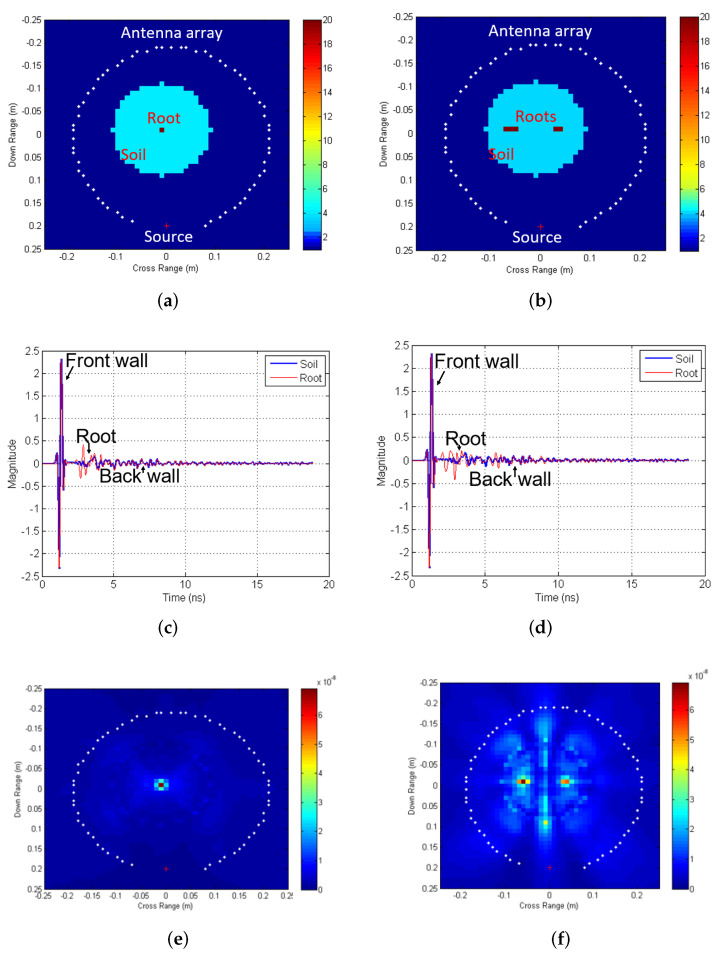
Time reversal simulation results for single and multiple root showing (**a**,**b**) model schematic with permittivity distribution for single and two roots. (**c**,**d**) Forward scattered signals for receiver 30 (highlighted with + in Figure 2a,b for single and two roots. (**e**,**f**) Time-integrated energy images detect the presence of roots for single and two root cases.

A 2D numerical study was conducted to validate the feasibility of the TR algorithm for root imaging applications. The electromagnetic wave equations were numerically modeled using a finite-difference time-domain (FDTD) algorithm. The overall schematic of a single root section and multiple root sections embedded in a soil region, along with a source antenna and a circular receiver antenna array is shown in Figure 2a,b. The soil region is assumed to comprise the moisture content with the dielectric constant ($\varepsilon_{r}$) = 10 and conductivity (σ) = 0.04 S/m at 6 GHz, while the root section is assumed to be mostly dry with $\varepsilon_{r}$ = 4 and σ = 0.008 S/m at 6 GHz.

A modulated Gaussian pulse with a width of 0.1 ns is used to excite the source antenna. The scattered fields are computed for this model and an equivalent model without the presence of the root to reduce the soil scattering effects and to obtain the root perturbations. The scattered field signals for Receiver 30, with and without the roots, for single and multiple roots are shown in Figure 2c,d. The received signals for the root model consist of the front wall scattering of the soil region followed by the root scattering, the back-wall scattering of the soil region and second order scattered fields.

The scattering due to the root is absent for the equivalent model without the root. The root perturbations for each receiver antenna are computed and numerically back-propagated using the FDTD TR algorithm. As seen in Figure 2e,f, the computed time-integrated energy shows efficient focusing around the root regions for both single and multiple roots. As can be seen in the case of multiple roots, cross-coupling between the two roots lead to some additional artifacts around the soil top and bottom interfaces in the energy images. The simulation results show that the proposed TR algorithm can effectively image buried roots in a soil environment.

### 2.2. HFSS Simulations

**Figure 3:**
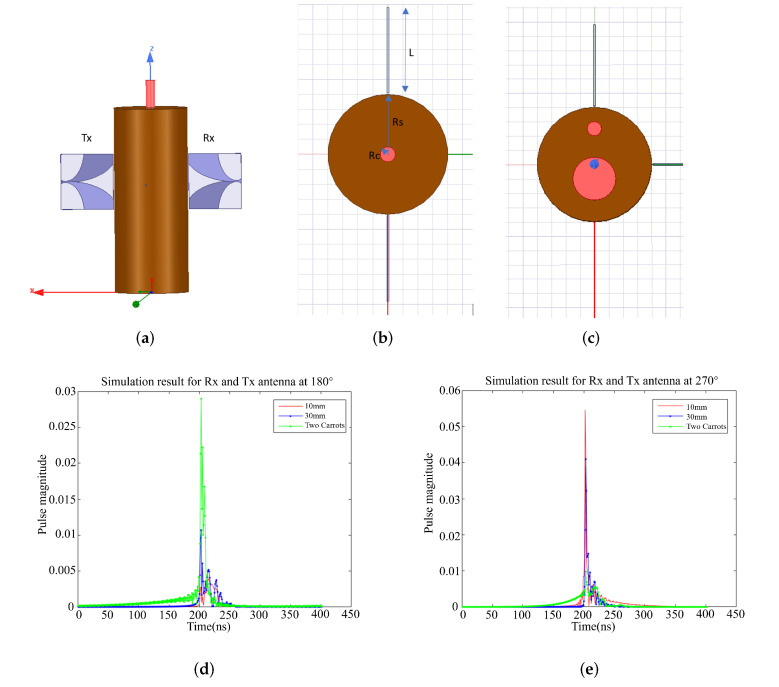
Schematic of the HFSS simulation showing (**a**) side view of a container with soil and a carrot and the location of the launcher and receiver antennas, (**b**) top view showing a container with soil and a single carrot and (**c**) top view showing a container with soil and two carrots. The time-domain results for the antennas at (**d**) 180${}^{\circ }$ and (**e**) 270${}^{\circ }$.

3D high-frequency structure simulation studies were conducted to analyze the sensitivity of the antenna system towards root detection using a commercial electromagnetic field simulator Ansys HFSS. An antipodal Vivaldi antenna was designed for ultra-wideband, high gain and beam symmetry to illuminate the root. The HFSS simulation model when the azimuth angle between Tx and Rx is 18${}^{\circ }$, is shown in Figure 3a–c as an illustration. The Tx is fixed and the Rx rotates around the scanned target root with a uniform angular step of 10${}^{\circ }$.

The start and endpoint for Rx are at an azimuth angle of 50${}^{\circ }$ from Tx. The radius of the soil (Rs) is 50 mm, and the radii of the root (Rc) are set to be 10 and 30 mm, respectively. The single carrot with different two radius sizes and the combination of these two carrots are all simulated to fully analyze the performance of the designed antenna as shown in Figure 3b,c.

The $S_{12}$ magnitude and phase frequency domain data are converted to time domain data using a standard inverse fast Fourier transform (IFFT) method. The envelopes of the pulses when the angular steps theta are 180${}^{\circ }$ and 270${}^{\circ }$ are shown in Figure 3d,e, respectively. The pulse magnitudes have a significant difference between the different radii of the carrots in every angular step. The larger the size of the root, the greater the pulse magnitude. In addition, the pulse magnitudes of two carrots are significantly different from that of a single carrot. The simulation results show that the proposed ultra-wideband microwave imaging system effectively identified different sizes and numbers of carrots buried in the soil.

## 3. Experimental Results

### 3.1. Experimental Setup

For the experimental setup, the antennas were connected to a vector network analyzer, which radiated power to the transmitter antenna and read the received power through the receiver Vivaldi.

**Figure 4:**
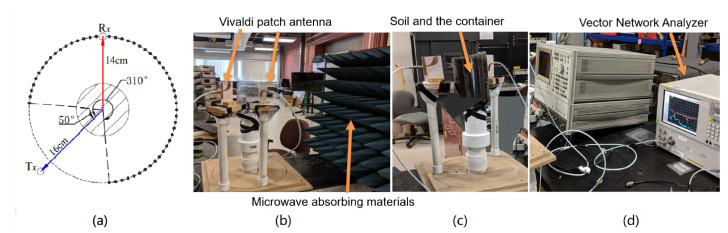
(**a**) Schematic of experimental setup showing the scanning location points spaced angularly at 10${}^{\circ }$ with Tx as transmitter and Rx as receiver. The experimental setup showing (**b**) Vivaldi-style patch antennas with a centrally located sample container containing soil and a carrot with a microwave absorber, (**c**) close view of soil container and two antenna supports of which one is mounted on a rotating base and (**d**) RF signal measurement equipment (VNA).

The experimental setup is shown in Figure 4. Two carrots of varying sizes were considered as samples under test. The average diameter of the small carrot was 16 mm, while that of the larger one was 25 mm. The diameter of the soil container was 100 mm. Microwave pyramidal foam absorber materials were placed in the surroundings of the setup to prevent spurious reflections from introducing error in the measurements.

Three sets of measurements (a small carrot, a large carrot and two carrots together) were performed to see the imaging capability of the system. The transmitter and receiver were placed at distances of 16 and 14 cm from the center of the container, respectively. The wood board shown in Figure 4b was used to accurately maintain the same relative distance between sensors and root. The starting position of the receiver subtended an angle of 50${}^{\circ }$ with the transmitter at the center of the container. A circumferential scan of 260${}^{\circ }$ (until the receiver is at 310${}^{\circ }$) was performed at steps of 10${}^{\circ }$. The experimental parameters are summarized in Table 2.

### 3.2. Results

**Figure 5:**
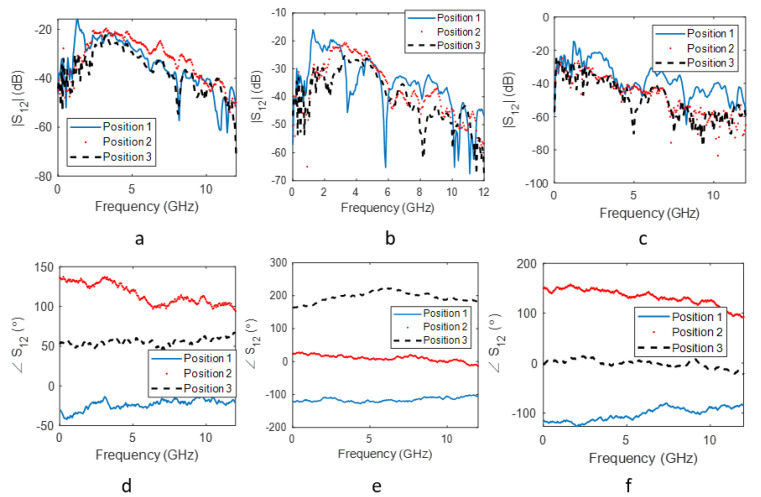
The insertion loss magnitude for (**a**) the small carrot, (**b**) the large carrot and (**c**) multiple carrots. The insertion loss phase for (**d**) the small carrot, (**e**) the large carrot and (**f**) multiple carrots.

Figure 5 shows the $S_{12}$ amplitude and phase for each group of experiments performed using the small carrot, large carrot and multiple carrots. Positions 1, 2 and 3 refer to the receiver positions at 90${}^{\circ }$, 180${}^{\circ }$ and 270${}^{\circ }$, respectively. We observed that the variations in magnitude were higher at 90${}^{\circ }$, while the phase change was similar at all three positions. However, the phase data for the three positions were more distinguishable than the amplitude data. This is because, even though the relative distance between the Tx and Rx did not change to a large extent, the angular change had a greater effect in the phase of the received signals.

**Figure 6:**
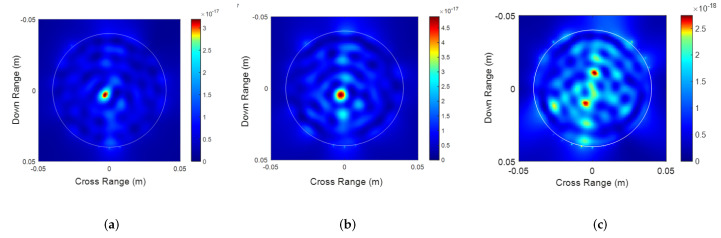
Time reversal imaging for (**a**) the small carrot, (**b**) the large carrot and (**c**) multiple carrots.

The frequency domain data was converted to time domain pulses and back propagated using an FDTD model. The images obtained by TR imaging for the three sets of measurements as shown in Figure 6a,b capture the change in sizes of the carrots. In the case of Figure 6c, we see two distinct localized spots corresponding to the two carrots. Additional artifacts are noticed more in the multiple carrots case. This can be attributed to the increase in multiple scattering when the waves encounter two roots instead of a single carrot.

### 3.3. Discussion on the Error Estimate

**Figure 7:**
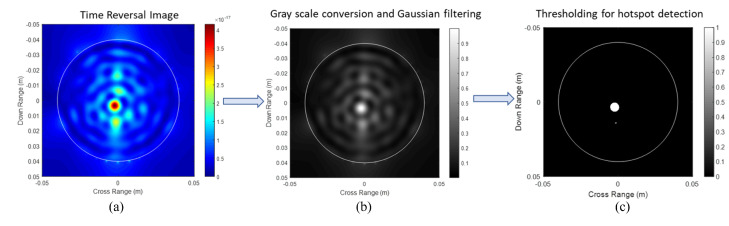
Image processing steps for the detection of targets. Root size and shape estimation of the large carrot (**a**) time reversal image, (**b**) image after gray scale conversion and Gaussian filtering and (**c**) thresholding for hotspot detection.

Figure 6a,b presents the individual TR images obtained for the 16 and 25 mm carrots, respectively. Figure 6a produces a cleaner image due to weaker scattering by the 16 mm carrot as compared to the 25 mm carrot. Estimation of the size of the target is done by applying image processing techniques on the TR images. Explicitly, the TR images are converted to gray-scale images and convolved with a Gaussian filter [3]. Appropriate thresholding is further done for the detection of hot-spots in the images, which correspond to the targets (roots in our case). We define an error metric ζ as:

(3) $$\zeta =\frac{y-x}{y}$$

where *y* is the true size of the target and *x* is the estimated size of the target obtained from TR. Figure 7a illustrates the image processing steps for the detection of the targets. This includes two steps where the first step converts the RGB image to a greyscale image and applies Gaussian filtering. The second step applies thresholding to detect the hotspot. The diameters of the targets are estimated to be 5 and 7 mm, respectively.

The shortest diameter of the hotspot is reported as the estimated size to avoid smudging caused due to experimental inaccuracies. The error metrics ζ were calculated to be 0.68 and 0.72, respectively, for 16 and 25 mm carrots. The differences can be attributed to several factors. Mainly, the source and receivers for the TR imaging are modeled in the FDTD code as point sources. However, in practice, they consist of patch antennas of finite sizes, and this approximation can cause localization errors, which in turn will result in an erroneous image target size. Any deviations in the modeled permittivity value of the soil (medium) from its actual value will cause an error in localization.

Although the exact localization spots do not correspond to their true sizes, it should be noted that the ratio of their sizes is equivalent, i.e., the small to large image target size ratio is 0.71, while the true size ratio is 0.64. Based on the results discussed above, the detection performance of the proposed root imaging technique in terms of sensitivity and accuracy is analyzed through simulation modeling-based study and experimental study. Both simulation and experiment results indicate that the developed sensing system can image single root and two roots cases clearly and differentiate the sizes of carrots with high contrast, which indicates that the proposed technique can identify buried root numbers in a soil environment efficiently.

Future work is being planned to involve machine learning on the signals or deep learning on the images generated to correct errors. The advantage of this method is the speed of imaging, which can be on the order of a few seconds depending on the scanning speed. With present day robotics technology, the scanning speed can be quite sufficient to achieve real-time imaging and evaluating the condition of roots in the field. The antenna parts can be made using rugged materials, like metals and Teflon, which are corrosion resistant and can withstand the harsh environment conditions endured by plants, like moisture and sunlight.

## 4. Conclusions

We proposed an ultra-wideband microwave imaging system for in situ nondestructive root phenotyping. The system was developed to estimate the location and shape of roots with soil background noise. The capability to provide the size and localization information of single and multiple roots demonstrated the simulation framework’s robustness.

Experiments using the developed Vivaldi patch antenna were conducted. Three cases, including a small carrot, a large carrot and two carrots, which were used to mimic different root conditions, were considered here, and the reconstruction results with precise and high imaging quality validated the proposed microwave imaging system’s accuracy. The 2D imaging can be improved to 3D phenotyping with the antenna array’s deployment both in the vertical and circumferential direction around the root for field implementation.

The implementation of the antenna array can fix the relative distance between sensors and the soil, which can help us to mitigate the influence of changes in the sensor location. The non-iterative TR algorithm proposed for signal processing was computationally efficient and enabled the rapid localization of roots. This work also shows its ability for the real-time monitoring of a root system in a real soil environment. The advantages of the rapid scanning ability and robustness enable the microwave imaging technique to be deployed in fields for scanning a large volume of soil and to access the state of the roots in a real-time manner.

## Figures and Tables

**Table 1:** Advantages and limits of the pre-existing root phenotype methods.

Method	Advantage	Limit
Excavation methods [11], pinboard method [12], trench profile technique [9], glass pane/tube method [13,14]	Easy and cost effective method	Destructive, time consuming, may affect and limit the growth of the root, low accuracy and low-resolution
Neutron radiography [15]	Provides root image	Destructive, inapplicable for in situ root phenotype
X-ray computed tomography [16,17,18,19,20]	Non-destructive, high resolution, high accuracy and fast 3D root phenotype	Expensive, destructive, non-portable, ineffective for in situ root phenotype
Magnetic resonance (MR) method [21,22]	Non-destructive, high accuracy and 3D root phenotype	Lower resolution and longer imaging time compared to the X-ray method. MR dependents on the water content of the root, and thus its accuracy may be influenced by the plant type and soil moisture
Laser root scanner [24]/confocal laser scanning microscopy [25]	Provide precise 3D measurements non-destructively	Destructive, can only be used when the root is growing in a transparent medium, requires longer imaging times, expensive
Cameras [26,27]	Provide precise images non-destructively and fast	Can only be used when the root is growing in a transparent medium
Fluorescence techniques [28,29]	Provide precise measurements	Destructive, can only be used when the root is growing in a transparent medium
Hyperspectral imaging method [9]	Discriminates between living, senescent and dead roots, leaf debris and soil	Can only be used when the root is growing in a transparent medium
THz imaging method [31]	High resolution images, detects and identifies roots and objects buried in soil	Scattering, absorption and radiation issues and unavailability of hardware for commercialization

**Table 2:** Experimental parameters.

Parameters	Values
Scanning angle	50–310${}^{\circ }$
Scanning step	10${}^{\circ }$
Vivaldi antenna frequency	3–10 GHz
Distance between receiver and soil container	14 cm
Distance between transmitter and soil container	16 cm
Diameter of soil container	100 mm
Diameter of carrots	16, 25 mm

## References

[1] Iyer-Pascuzzi A.S., Symonova O., Mileyko Y., Hao Y., Belcher H., Harer J., Weitz J.S., Benfey P.N. (2010). Imaging and analysis platform for automatic phenotyping and trait ranking of plant root systems. Plant Physiol..

[2] Paustian K., Campbell N., Dorich C., Marx E., Swan A. (2016). Assessment of Potential Greenhouse Gas Mitigation from Changes to Crop Root Mass and Architecture.

[3] Liu J., Ren Y. (2020). A general transfer framework based on industrial process fault diagnosis under small samples. IEEE Trans. Ind. Inform..

[4] Fu M., Liu J., Zhang H., Lu S. (2020). Multisensor Fusion for Magnetic Flux Leakage Defect Characterization Under Information Incompletion. IEEE Trans. Ind. Electron..

[5] Luo L., Liu W., Lu Q., Wang J., Wen W., Yan D., Tang Y. (2021). Grape berry detection and size measurement based on edge image processing and geometric morphology. Machines.

[6] Wu F., Duan J., Chen S., Ye Y., Ai P., Yang Z. (2021). Multi-target recognition of bananas and automatic positioning for the inflorescence axis cutting point. Front. Plant Sci..

[7] Chen M., Tang Y., Zou X., Huang Z., Zhou H., Chen S. (2021). 3D global mapping of large-scale unstructured orchard integrating eye-in-hand stereo vision and SLAM. Comput. Electron. Agric..

[8] Tracy S.R., Nagel K.A., Postma J.A., Fassbender H., Wasson A., Watt M. (2020). Crop Improvement from Phenotyping Roots: Highlights Reveal Expanding Opportunities. Trends Plant Sci..

[9] Bodner G., Nakhforoosh A., Arnold T., Leitner D. (2018). Hyperspectral imaging: A novel approach for plant root phenotyping. Plant Methods.

[10] Clark R.T., MacCurdy R.B., Jung J.K., Shaff J.E., McCouch S.R., Aneshansley D.J., Kochian L.V. (2011). Three-Dimensional Root Phenotyping with a Novel Imaging and Software Platform. Plant Physiol..

[11] Smit A.L., Bengough A.G., Engels C., van Noordwijk M., Pellerin S., van de Geijn S.C. (2013). Root Methods: A Handbook.

[12] Schuurman J., Goedewaagen M. (1965). Methods for the Examination of Root Systems and Roots.

[13] Böhm W. (2012). Methods of Studying Root Systems.

[14] Bates G. (1937). A device for the observation of root growth in the soil. Nature.

[15] Moradi A.B., Conesa H.M., Robinson B., Lehmann E., Kuehne G., Kaestner A., Oswald S., Schulin R. (2009). Neutron radiography as a tool for revealing root development in soil: Capabilities and limitations. Plant Soil.

[16] Gregory P.J., Hutchison D., Read D.B., Jenneson P.M., Gilboy W.B., Morton E.J. (2003). Non-invasive imaging of roots with high resolution X-ray micro-tomography. Roots: The Dynamic Interface between Plants and the Earth.

[17] Tracy S.R., Roberts J.A., Black C.R., McNeill A., Davidson R., Mooney S.J. (2010). The X-factor: Visualizing undisturbed root architecture in soils using X-ray computed tomography. J. Exp. Bot..

[18] Schmidt S., Bengough A.G., Gregory P.J., Grinev D.V., Otten W. (2012). Estimating root–soil contact from 3D X-ray microtomographs. Eur. J. Soil Sci..

[19] Atkinson J.A., Pound M.P., Bennett M.J., Wells D.M. (2019). Uncovering the hidden half of plants using new advances in root phenotyping. Curr. Opin. Biotechnol..

[20] Downie H.F., Adu M., Schmidt S., Otten W., Dupuy L.X., White P., Valentine T.A. (2015). Challenges and opportunities for quantifying roots and rhizosphere interactions through imaging and image analysis. Plant Cell Environ..

[21] Metzner R., Eggert A., Van Dusschoten D., Pflugfelder D., Gerth S., Schurr U., Uhlmann N., Jahnke S. (2015). Direct comparison of MRI and X-ray CT technologies for 3D imaging of root systems in soil: Potential and challenges for root trait quantification. Plant Methods.

[22] Pflugfelder D., Metzner R., van Dusschoten D., Reichel R., Jahnke S., Koller R. (2017). Non-invasive imaging of plant roots in different soils using magnetic resonance imaging (MRI). Plant Methods.

[23] Wasson A.P., Nagel K.A., Tracy S., Watt M. (2020). Beyond Digging: Noninvasive Root and Rhizosphere Phenotyping. Trends Plant Sci..

[24] Fang S., Yan X., Liao H. (2009). 3D reconstruction and dynamic modeling of root architecture in situ and its application to crop phosphorus research. Plant J..

[25] Turillazzi E., Karch S.B., Neri M., Pomara C., Riezzo I., Fineschi V. (2008). Confocal laser scanning microscopy. Using new technology to answer old questions in forensic investigations. Int. J. Leg. Med..

[26] Tsaftaris S.A., Noutsos C. (2009). Plant phenotyping with low cost digital cameras and image analytics. Information Technologies in Environmental Engineering.

[27] Fahlgren N., Gehan M.A., Baxter I. (2015). Lights, camera, action: High-throughput plant phenotyping is ready for a close-up. Curr. Opin. Plant Biol..

[28] Faget M., Blossfeld S., Von Gillhaußen P., Schurr U., Temperton V.M. (2013). Disentangling who is who during rhizosphere acidification in root interactions: Combining fluorescence with optode techniques. Front. Plant Sci..

[29] Watt M., Hugenholtz P., White R., Vinall K. (2006). Numbers and locations of native bacteria on field-grown wheat roots quantified by fluorescence in situ hybridization (FISH). Environ. Microbiol..

[30] Singhvi A., Ma B., Scharwies J.D., Dinneny J.R., Khuri-Yakub B.T., Arbabian A. Non-Contact Thermoacoustic Sensing and Characterization of Plant Root Traits. Proceedings of the 2019 IEEE International Ultrasonics Symposium (IUS).

[31] Smith N., Rivera L.A., Burford N., Bowman T., El-Shenawee M.O., DeSouza G.N. Towards root phenotyping in situ using THz imaging. Proceedings of the 2015 40th International Conference on Infrared, Millimeter, and Terahertz Waves (IRMMW-THz).

[32] Mukherjee S., Shi X., Deng Y., Udpa L. (2020). A Hybrid Microwave NDE System for Rapid Inspection of GFRP Composites. Mater. Eval..

[33] Rathod V.T., Banerjee P., Deng Y., Ida N., Meyendorf N. (2019). Low Field Methods (GMR, Hall Probes, etc.). Handbook of Advanced Non-Destructive Evaluation.

[34] Shi X., Rathod V.T., Mukherjee S., Udpa L., Deng Y. (2020). Multi-modality strain estimation using a rapid near-field microwave imaging system for dielectric materials. Measurement.

[35] Kazemi N., Schofield K., Musilek P. (2021). A high-resolution reflective microwave planar sensor for sensing of vanadium electrolyte. Sensors.

[36] Abdolrazzaghi M., Daneshmand M., Iyer A.K. (2018). Strongly enhanced sensitivity in planar microwave sensors based on metamaterial coupling. IEEE Trans. Microw. Theory Tech..

[37] Herrmann P.S.d.P., Sydoruk V., Marques Porto F.N. (2020). Microwave Transmittance Technique Using Microstrip Patch Antennas, as a Non-Invasive Tool to Determine Soil Moisture in Rhizoboxes. Sensors.

[38] Menzel M.I., Tittmann S., Buehler J., Preis S., Wolters N., Jahnke S., Walter A., Chlubek A., Leon A., Hermes N. (2009). Non-invasive determination of plant biomass with microwave resonators. Plant Cell Environ..

[39] Mukherjee S., Udpa L., Deng Y., Chahal P., Rothwell E.J. (2017). Design of a microwave time reversal mirror for imaging applications. Prog. Electromagn. Res..

[40] Lerosey G., De Rosny J., Tourin A., Derode A., Montaldo G., Fink M. (2004). Time reversal of electromagnetic waves. Phys. Rev. Lett..

[41] Mukherjee S., Udpa L., Udpa S., Rothwell E.J. (2016). Target localization using microwave time-reversal mirror in reflection mode. IEEE Trans. Antennas Propag..

[42] Mukherjee S., Tamburrino A., Haq M., Udpa S., Udpa L. (2018). Far field microwave NDE of composite structures using time reversal mirror. NDT E Int..

